# Spina bifida occulta in children: presentation and outcome

**DOI:** 10.1186/1743-8454-7-S1-S9

**Published:** 2010-12-15

**Authors:** Margareta Dahl, Gunnar Ahlsten

**Affiliations:** 1 Department of Women’s and Children’s Health, Uppsala University Hospital 751 85, Uppsala, Sweden

## Background

Occult spinal dysraphism (OSD) encompasses a wide spectrum of congenital anomalies. Since OSD can lead to irreversible neurological, urological and orthopedic dysfunction, early diagnosis and treatment is necessary. In this study we evaluated a selected cohort of patients with OSD with regard to presenting symptoms and clinical outcome. The main purpose of this project is to develop clinical guidelines for this condition.

## Materials and methods

We retrospectively reviewed the medical records of all children with OSD referred to University Hospital Uppsala during the period 2000-2008. Fifty-six Children (22 males and 34 females) aged from 3 days to 16 years were identified having OSD. All had MR scans carried out. The median observation time was 11 years.

## Results

The first sign of neurological dysfunction was noticed at the median age of two years. The symptoms included deformities of feet, bladder dysfunction and gait disturbance. Retrospectively, cutaneous lesions in the back were found in 43 children. The MR scans demonstrated in all children various congenital anomalies such as diastematomyelia or lipomyelomeningocele. During the follow-up period deterioration was seen in approximately half of the patients. Twenty-eight children underwent detethering operation. Despite this operation the symptoms of eight of these children progressed. At follow-up at the median age of 11 years 38 children had clinical signs and symptoms of neurological dysfunction whereof 19 children had severe persistent symptoms.

## Conclusions

In this study one third of the children with OSD developed persistent severe symptoms of neurological dysfunction. It is highly important to detect these lesions before the occurrence of neurological or urological manifestations. Urinary incontinence is often the first clinical symptom, but can be difficult to evaluate when the child is very young.

